# Rural-to-urban migration and its implication for new cooperative medical scheme coverage and utilization in China

**DOI:** 10.1186/1471-2458-11-520

**Published:** 2011-06-30

**Authors:** Peiyuan Qiu, Yang Yang, Juying Zhang, Xiao Ma

**Affiliations:** 1 West China School of Public Health, Sichuan University, No. 17, Section 3, South Renmin Road, Chengdu, Sichuan, China

**Keywords:** rural-to-urban, migration, New Cooperative Medical Scheme, NCMS, China

## Abstract

**Background:**

China has been experiencing the largest rural to urban migration in history. Rural-to-urban migrants are those who leave their hometown for another place in order to work or live without changing their *hukou *status, which is a household registration system in China, categorizing people as either rural residents or urban residents. Rural-to-urban migrants typically find better job opportunities in destination cities, and these pay higher salaries than available in their home regions. This has served to improve the enrollment rates in the New Cooperative Medical Scheme (NCMS) of rural families, protecting households from falling into poverty due to diseases. However, current regulations stipulate that people who are registered in China's rural *hukou *can only participate in their local NCMS, which in turn poses barriers when migrants seek medical services in the health facilities of their destination cities. To examine this issue in greater depth, this study examined the associations between migration, economic status of rural households, and NCMS enrollment rate, as well as NCMS utilization of rural-to-urban migrants.

**Methods:**

A multistage cluster sampling procedure was adopted. Our sample included 9,097 households and 36,720 individuals. Chi-square test and T-test were used to examine differences between the two populations of migrants and non-migrants based on age, gender, marriage status, and highest level of education. Ordinal logistic regression was used to examine the association between migration and household economic status. Binary logistic regression was used to examine the associations between household economic status, migration and enrollment in the NCMS.

**Results:**

Migration was positively associated with improved household economic status. In households with no migrants, only 11.3% of the population was in the richest quintile, whereas the percentage was more than doubled in households with family members who migrated in 2006. Among those using in-patient medical services, 54.3% of migrants in comparison with 17.5% of non-migrants used out-of-county hospitals, many of which were not designated hospitals (Designated hospitals refer to hospitals where, if people use in patient health care, could receive reimbursement from the NCMS.); and 55.2% of migrants in comparison with 24.6% of non-migrants, who had the NCMS in 2006, received no reimbursement from the NCMS. The three main reasons of not receiving reimbursement were: staying in a hospital not designated by the NCMS, lack of knowledge of NCMS policies, and encountering difficulties obtaining reimbursement.

**Conclusion:**

Migrants to urban centers improve the economic status of their rural household economic of origin. However, obtaining reimbursement under the current NCMS for the cost of hospital services provided by undesignated providers in urban centers is limited. Addressing this challenge is an emerging policy priority.

## Background

Medical debt can prevent rural households from moving out of poverty or can drive families into poverty [1]. In China, the central government has been striving to create universal medical insurance. Currently, the country has three primary health insurance programs, namely, the Urban Employee Basic Medical Insurance (UEBMI) for the urban employed, the Urban Resident Basic Medical Insurance (URBMI) for urban residents, and the New Cooperative Medical Scheme (NCMS) for rural residents. The NCMS was initiated in rural China in 2003, which is a scheme of voluntary mutual assistance among participating rural residents improving access to health care services and protecting against catastrophic illnesses [2,3]. The NCMS is financed in China's poorer central and western regions by a combination of contributions from the central government, local governments, and individuals. In 2006, at the time of our sampling, annual contributions amounted to RMB20 (Chinese yuan) per insured person from the central government, RMB15-20 from the local governments, and RMB10-15 from each insured individual [4]. (By 2010, these had increased to RMB60, RMB60, and RMB30, respectively, and the premium paid by rural residents in households identified as "poor" (about 5% of total households) has been waived [5,6].) The unit of enrollment is at household level in order to avoid adverse selection within the household [7]. National guidelines of the NCMS focus on the coverage of in-patient care, while some provinces have been in fact developing a benefit package covering both outpatient and inpatient services. Reimbursement rates differ, with higher rates (35%-60%) at rural township health care centers and lower rates (25%-40%) at county-level facilities. Because funding for the NCMS is determined at the county level, it commonly requires participants to use designated facilities within the county, which often are county-level hospitals and township health care centers. Of note, and particularly important for this study, while NCMS plans pay for services provided outside of the home counties [8], the reimbursement rates are discounted sharply (10%-25%). According to NCMS regulations, the ceiling level of reimbursement in 2006 was RMB10,000-20,000 per participant per year. (This was increased to RMB50,000-100,000 per participant per year by 2010.)

Previous research mainly focused on impact evaluation on the NCMS, willingness to join the NCMS, equality of the NCMS, and adverse selection in the NCMS [7,9-13]. Nevertheless, little empirical research has been done on the associations between rural-to-urban migration and NCMS coverage and utilization in rural China. Rural-to-urban migrants are those who leave their hometown for another place in order to work or live without changing their *hukou *status. *Hukou *is a household registration system in China [14], categorizing people as either rural residents or urban residents. When strictly enforced in past times, one's *hukou *defined where an individual could live and work [14,15]; even now it affords different rights to migrants and local residents. For decades, *hukou *has functioned like an "internal passport system" [16]. A study among rural-to-urban migrants in Indonesia found that migrants were more likely to have insurance coverage, because health insurance in Indonesia was made available primarily through urban employers, which resulted in a higher utilization of medical care among migrants [17]. China, with a large population of internal rural-to-urban migrants, has a different story.

Since the mid-1980s, migration in China has been economically driven. Residents from less developed rural regions in western and central China traveled to developed cities and eastern industrial zones ("urban centers") to seek better job opportunities and higher incomes. They typically send remittances to their families, providing financial support, while returning to their homes for holidays and occasionally for helping with the harvest. Based on data from National Bureau of Statistics of China, 10.0% of migrant workers sent 30%-40% of their income to their families; 15.3% sent 40%-50% of earnings; 11.2% sent 50%-60%; 23.5% sent 60%-70%; and 9.2% sent 70%-80% of their income home [18].

These rural-to-urban migrant workers have made a tremendous contribution to China's economic development and played an important role in financial safety of their families. The NCMS is designed exclusively for rural people, according to their *hukou*. Funding of the NCMS is based on the county level of governmental organization; therefore, it is expected that people will seek medical services in designated hospitals, most of which are located within the home county. Nevertheless, this is impractical for migrant workers, limiting how much they can use their NCMS benefits. At the same time, this population faces a dilemma in terms of access to health insurance in cities. Rural-to-urban migrants always are excluded from city health systems, so that most of them cannot qualify for the UEBMI and URBMI, as if they were local city residents, even when they are working in the same company and living in the same community [19]. But as they are out-of-county, reimbursement for city services is severely limited.

In this study we examined the associations between migration and household economic status, enrollment in the NCMS, and use of its benefits. Specifically, we anticipated that migration had a positive association with household economic status, which may in turn improve the enrollment rate in the NCMS of families potentially. However, since people who hold rural *hukou *are expected to participate in and get reimbursement from the NCMS in the location of their *hukou*, migrants in urban centers confront difficulty when seeking medical services in the health facilities of their destination cities [2].

In turn, this lack of meaningful "insurance portability" eventually may lead to a lower long-term enrollment in the NCMS by premium-paying migrants, thus jeopardizing the viability of the system in the future. To date, we know of no related empirical research regarding these issues, although some authors have discussed challenges, such as a low reimbursement rate associated with the NCMS for migrants [20].

## Methods

### Key concepts

A migrant in the study is defined as an individual who moved in 2006 from the place of his/her rural *hukou *to another place in order to work or live without a change in *hukou*. A minimum of six months in residence away from home was required to qualify.

We defined persons suffering major illness as those who either accepted hospital treatment at least once or spent a minimum of RMB1000 on outpatient services, or experienced a diagnosed disability in 2006.

### Sources of data

A household survey was undertaken in two purposively selected counties in Sichuan Province in the Southwestern China and another two in Hubei Province in Central China as part of the program "Protecting the rural poor against the economic consequences of major illness: A challenge for Asian transitional economies (POVILL)". With a large number of rural residents, Sichuan and Hubei are two of the underdeveloped provinces in China and also major provinces to export labor. Inclusion criteria for counties included being a NCMS pilot county (except for the control county), and being a national-level poor county. In each area, a multistage cluster sampling procedure was adopted to select village communities, each consisting of around 100 households. All households in these communities were enumerated, giving a total sample of around 12,000 households. In this study, we recruited three of the four counties that had implemented the NCMS in 2006, yielding a sample of 9,097 households and 36,720 individuals (Table 1). Professors and graduate students from West China School of Public Health, Sichuan University and from Zhongnan University of Economics and Law were trained to conduct household surveys.

**Table 1:** Basic information of the three counties


**Indicators**	**Hongan, Hubei**	**Langzhong, Sichuan**	**Fushun, Sichuan**

Total population (N)	650,000	860,000	102,000,0
Rural population (N)	540,000	600,000	830,000
Average annual income of rural population (RMB/capital year)	2,328	2,889	3,400
Number of households involved in the study (N)	3,043	2,969	3,085
Number of individuals involved in the study (N)	12,712	11,079	12,929

### Data collection

A questionnaire developed for this study was used to interview the target population. The questionnaire has five sections. Sections A and E were used to collect household data on economic status, enrollment status in the NCMS, and utilization of the NCMS. Sections B, C and D were designed to obtain individual information on socio-demographic characteristics, health care demand and utilization by those who were sick in the past 14 days and health care demand and utilization by people who suffered major illness in 2006. Informed consent was obtained from all participants following a protocol that was approved by Ethics Committee of Sichuan University.

The household head or the person who knew family issues best was asked to answer all of the five sections and other family members were asked to finish Sections B, C and D. For those who were not at home when the interview was conducted, family members helped to provide their information.

### Asset index construction

Household income and expenditure are usually used as a tool for classifying household economic status. However, several investigators have noted potential limitations of this measure [21,22]. The quality of income and expenditure data may be questionable. These data are collected on the basis of personal recall and are prone to measurement errors. Problems of inaccurate reporting of income and expenditure, and difficulties in converting household products into monetary terms also serve as concerns.

**Figure 1:**
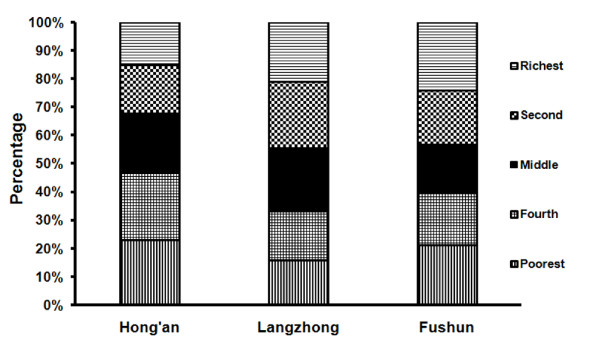
**The distribution of household economic status by county**. An asset index was constructed by using principal component analysis (PCA) to classify household economic status. Eleven variables from Section A of the questionnaire were used to calculate the PCA (Appendix A). The first principal component (eigenvalue: 3.315, account for 30.14%) was used to quintile the household economic status (Appendix B). As a result, each household was assigned into one of the five quintiles.

Thus, an asset-based index was introduced and developed as an alternative tool for classifying household economic status [21-24]. Considering that rural people in China usually have household products, which are very difficult to monetize, we constructed an asset index by using principal component analysis (PCA). Eleven variables from Section A of the questionnaire were used to calculate the PCA (Appendix A). The first principal component (eigenvalue: 3.315, account for 30.14%) was used to quintile the household economic status (Appendix B). As a result, each household was assigned into one of the five quintiles (Figure 1).

### Data analysis

Sociodemographic characteristics of migrants and non-migrants, including age, gender, highest education level, and marital status were described. Chi-square test and T-test were used to examine differences between the two populations of migrants and non-migrants based on these characteristics.

Household economic status and NCMS enrollment rates in the households with different migration characteristics were described first. Ordinal logistic regression was used to examine the association between migration and household economic status. The dependent variable was the quintile economic status using 1 = richest, 2 = second, 3 = middle, 4 = fourth, and 5 = poorest. Independent variables included household inactive labor force rate, household migration rate, household gender ratio, household head's education level, household disease burden, and household size.

(1) Household inactive labor force rate = the number of people not working in a household/household size

(2) Household migration rate = the number of migrants in a household/household size

(3) Household gender ratio = the number of males in a household/household size

(4) Household head's education level was defined as 1 = illiteracy, 2 = primary school, 3 = middle school, and 4 = high school and above

(5) Household disease burden = the number of people with a major illness/household size

(6) Household size = the number of people who were under the same *hukou*

Binary logistic regression was used to examine the relationships between household economic status, migration and enrollment in the NCMS. The dependent variable was whether a household was participating in the NCMS in 2006, where 0 = non-enrollment and 1 = enrollment. Independent variables included household economic status, household migration rate, household head's education level, household disease burden, and household size. Statistical significance was defined as *P *≤ 0.05.

In addition, reimbursement rates, type of health care institutions where to seek inpatient care, and reasons for not receiving NCMS reimbursement for non-migrants and migrants who had received inpatient services in 2006 were described.

## Results

### Sample description

The sample generated in our investigation consisted of a total of 9,097 households and 36,720 individuals. Because migration status was missing for 260 individuals, the sample for this analysis consisted of 9,018 households and 36,460 individuals. Table 2 presented the distributions of the study variables for the valid sample. In this sample: 34.3% of the population were migrants. Migrants were younger than non-migrants (*t *= 62.801, *P *< 0.001). The mean age was 42.7 years for non-migrants and 28.9 years for migrants. The data indicated that much more males than females migrated (60% vs. 40%, *χ*^2 ^= 441.876, df = 1, *P *< 0.001). Marital distribution between non-migrants and migrants was different (*χ*^2 ^= 1346.186, df = 3, *P *< 0.001), with 28.4% unmarried among non-migrants and 40.2% among migrants. Education level between non-migrants and migrants also differed significantly (*χ*^2 ^= 6735.647, df = 5, *P *< 0.001). Compared with non-migrants, more migrants had completed middle school or more education (62.1% vs. 25.9%).

**Table 2:** Demographic characteristics of non-migrants and migrants


		**Non-migrants**	**Migrants**^**a**^

Age (S.D)		42.7(23.4)	28.9(10.5)
			***t *= 62.801, *P *< 0.001**
Gender (%)	Male	48.4	60.0
	Female	51.6	40.0
			*χ*^2 ^**= 441.876, df = 1, *P *< 0.001**
Marriage Status (%)	Unmarried	28.4	40.2
	Married	62.9	58.4
	Divorced	0.3	0.9
	Widowed	8.4	0.5
			*χ*^2 ^**= 1346.186, df = 3, *P *< 0.001**
Highest level of education (%)	Illiteracy	36.7	3.2
	Primary school	37.4	29.8
	Middle school	20.5	49.2
	High school	5.1	12.9
	College and above	0.3	4.9
			*χ*^2 ^**= 6735.647, df = 5, *P *< 0.001**

a. Migrant here is defined as an individual who move from the place where his/her *hukou *is to another place for at least six months in order to work or live in 2006.

### Associations between migration, household economic status, and enrollment in the NCMS

Table 3 showed that 11.3% of the population in households with no migrants was in the richest quintile; whereas the percentages were 23.9% and 23.2% for households with half or fewer and more than half of family members migrating in 2006, respectively. Meanwhile, in households with no migrants, 34.3% of the population was classified into the poorest quintile, compared with 13.6% and 15.0% in households with half or fewer and more than half of family members migrating in 2006, respectively. Households with half or fewer members migrating in 2006 had an enrollment rate of 90.4%, and households with more than half of its members as migrants had an enrollment rate of 86.9%. Enrollment rates of richest, second, middle, fourth and poorest quintiles were 92.0%, 91.6%, 90.7%, 89.2%, and 83.9%, respectively. The poorest had the lowest enrollment rate.

**Table 3:** Household economic status and enrollment in the NCMS of households with different migration characteristics


		**Households with different migration characteristics**			
		
		**Households with no migrants (%)**	**Households with half or fewer family members migrating in 2006 (%)**	**Households with more than half of family members migrating in 2006 (%)**	**Total (%)**

Household economic status	Richest	11.3	23.9	23.2	20.0
	Second	14.7	22.6	20.7	20.0
	Middle	17.7	20.3	23.6	20.0
	Fourth	21.9	19.6	17.5	20.0
	Poorest	34.3	13.6	15.0	20.0
	Total	100.0	100.0	100.0	100.0
					
Did you join the NCMS?	Yes	89.2	90.4	86.9	89.5
	No	10.8	9.6	13.1	10.5
	Total	100.0	100.0	100.0	100.0

The ordinal logistic regression model (Table 4) showed that factors positively associated with household's economic status were lower inactive labor force rate, higher household migration rate, higher education level of household head, lower household disease burden and larger household size. Analysis of the results indicated that after controlling for household size, inactive labor force rate, household head's education level, household disease burden, and household gender ratio, migration still had a positive association with household economic status.

**Table 4:** Results of ordinal logistic regression on household economic status


	**Household economic status (1 = richest, 2 = second, 3 = middle, 4 = fourth, and 5 = poorest)**	
	
	**OR (95% CI)**	***P***

Household unemployment rate	2.334 (1.999,2.723)	**< 0.001**
Household migration rate	0.687 (0.582,0.813)	**< 0.001**
Household head's education level	0.925 (0.876,0.977)	**0.005**
Household gender ratio	1.201 (0.962,1.499)	0.105
Household disease burden	1.617 (1.450,1.804)	**< 0.001**
Household size	0.721 (0.701,0.741)	**< 0.001**

In the binary logistic regression model (Table 5), a household was more likely to enroll in the NCMS if household economic status was higher, household migration rate was lower and household size was larger. Relationships between enrollment rate and household head's education level, as well as gender ratio of a household were not significant. The results indicated that increased household economic status may improve enrollment rate of the NCMS, but higher household migration rate may decrease enrollment rate.

**Table 5:** Results of binary logistic regression on enrollment in the NCMS


	**Enrollment of the NCMS (0 = non-enrollment and 1 = enrollment)**	
	
	**OR (95% CI)**	***P***

Household migration rate	0.402 (0.298,0.543)	**< 0.001**
Household head's education level	0.938 (0.845,1.041)	0.225
Household disease burden	1.120 (0.926,1.355)	0.242
Household economic status	0.828 (0.786,0.873)	**< 0.001**
Household size	1.139 (1.081,1.200)	**< 0.001**

### Access to hospital care and reimbursement of service expenses by non-migrants and migrants

Out of those using in-patient services, 54.3% of migrants in comparison with 17.5% of non-migrants used out-of-county hospitals, many of which were not designated hospitals; and 55.2% of migrants in comparison with 24.6% of non-migrants, who had the NCMS in 2006, received no reimbursement from the NCMS and non-migrants received higher NCMS reimbursement rates than migrants (*Z *= -9.239, *P *< 0.001) (Table 6). Average reimbursement rates of local township health care centers, local county level hospitals, and out-of-town hospitals were 23.6%, 15.7%, and 4.4%, respectively. Three primary reasons accounted for lower reimbursement rates, although they contributed differently to the difficulties encountered by non-migrants and migrants: 1) Staying in a hospital that is not designated by the NCMS: non-migrants, 33.6%; migrants, 64.9%; 2) lack of knowledge of NCMS policies: non-migrants, 27.3%; migrants, 12.2%; and 3) encountering difficulties obtaining reimbursement: non-migrants, 18.6%; migrants, 12.8%.

**Table 6:** Use of hospital care by type, NCMS reimbursement rate, and reasons of not receiving NCMS reimbursement by non-migrants and migrants receiving inpatient services in 2006


		**Non-migrants**	**Migrants**
		
		**%**	**%**

Use of hospital care by type	Local township health care centers	39.0	17.8
	Local county level hospitals	43.5	27.9
	Out-of-county hospitals (city level and above hospitals, private hospitals)	17.5	54.3
	Total	100.0	100.0
NCMS reimbursement rates^a^	0%	24.6	55.2
	> 0% and ≤ 10%	16.0	9.3
	> 10% and ≤ 20%	18.1	16.0
	> 20% and ≤ 30%	19.2	8.6
	> 30% and ≤ 40%	15.0	7.1
	> 40%	7.0	3.7
	Total	100.0	100.0
Reasons of not receiving NCMS reimbursement after inpatient services^a^	Deductible fee is higher than inpatient services expenditure	8.6	4.1
	Most of the expenses could not be covered by the NCMS	9.5	2.7
	Stay in a hospital that is not designated by the NCMS	33.6	64.9
	Lack of knowledge of the NCMS policies	27.3	12.2
	Encountering difficulties obtaining reimbursement	18.6	12.8
	Others^b^	2.3	3.4
	Total	100.0	100.0

a. Only people who had the NCMS in 2006 were included in the analysis.

b. Others include other people paying for the inpatient expenses, and other insurance paying for inpatient expenses

## Discussion

### Rural-to-urban migration and NCMS coverage and utilization in China

Sichuan Province and Hubei Province are two of the largest sources of rural-to-urban migrants. In this study, 34.3% of the population were migrants. As showed in Table 2, migrants, as compared with non-migrants, tend to be younger men having higher education level. Because they are younger, the unmarried proportion is larger. The results are in accordance with previous studies [25,26].

While internal rural-to-urban migration during the past two decades has fueled China's economic transformation, in particular supplying an essential workforce for its export economy and the growth of its cities, it also has served as a key element in improving the economic circumstances of rural regions. People migrate to cities for better job opportunities and higher incomes, and to assist their families economically. Though many migrants only return home for the Traditional Spring Festival, their remittances may be the primary source of household income, providing an important financial safety net against the risks of crop failure, ill health, or other shocks to rural households (e.g., weather related). The National Bureau of Statistics of China reported that, 43.8% of migrant workers sent more than 50% of their income back home in 2006 [21].

In a related vein, Tan found among rural households from 1985 to 2007, the proportion of salaried income, as a component of the total household earnings, increased from 18.0% to 38.6% [27], underscoring the diminishing relative role of agrarian-generated income. Our study found that 34.3% of households without migrants were included in the poorest quintile. For households where half or fewer members were migrants, this dropped to 23.9%, and for those with more than half as migrants, 23.2% were in the poorest quartile. Although we detected a positive association between migration and household economic status, we also recognize the potential limitation of our cross-sectional design, where it was not possible to determine pre-migration household economic status.

Household economic status was found to be positively associated with enrollment rate in the NCMS; however, we detected that, while rural households in general have a higher enrollment in the NCMS, the enrollment rate in 2006 was slightly lower for those where more than half of the family members worked as migrants. Competing forces may be influencing these findings: 1) The participation unit is a household rather than an individual, which tends to avoid the effect of adverse selection [13], especially as migrants themselves tend to be younger and in good health; 2) migration may improve household economic status, relieving the burden of NCMS premium and promoting a higher enrollment rate; 3) given the very limited benefits eligibility of migrants working in cities far from their *hukou*, household having many migrant members may choose not to participate in the NCMS in the future.

In fact, our data revealed that seeking hospital care in out-of-county hospitals resulted in a much lower reimbursement rates or even no reimbursement from the NCMS. However, 54.3% of migrants chose hospital care in out-of-town hospitals, usually where they worked and lived. As a result, a high proportion of migrants (55.2%) who used inpatient services in 2006 received no NCMS reimbursement from for these services.

National policy has long been established on locality-based schemes that depend on *hukou *as an organizing principle: Like many initiatives, the NCMS continues to be financed and administered by county governments, and understandably requires local enrollees to use designated facilities within the county. This is likely to be exacerbated by the greater costs of urban (better equipped and better staffed) medical centers in comparison to the costs associated with China's modest town hospitals. Even for the few NCMS plans that accept medical bills from urban hospitals [8], the level of payment is extremely low and the procedures for reimbursement to individuals often are long, cumbersome, and unpleasant. In one of our study sites, Hong'an County, NCMS plan reimburses participants for 50% of the inpatient services cost in a township health care center, 35%-50% of that in a county hospital, and 12.5-20% in a hospital out of the home county (2006 rates). When migrants do receive out-of-county services, they must inform their hometown health department before service delivery, as well as provide proof of employment in the city. The other two counties included in our study have similar procedures.

An additional system-level challenge may relate to the newness of the NCMS insurance scheme. According to the "Funding Regulation on the New Cooperative Medical Scheme" published by the Ministry of Finance and Ministry of Health [28], surplus funds at the end of the year should not exceed 15% of revenue, and the cumulative surplus should be remain less than 25%. During the initial phases of implementation, many NCMS management teams were concerned about exhausting annual revenues and worked vigorously to build reserves. A case study on the financial management of the NCMS in six counties in two Chinese provinces found that five out of six held a large fund surplus, while their enrollees obtained only partial financial protection [29]. In order to reduce schemes surpluses, some counties will do a second round reimbursement usually at the end of a year. In our sample counties, the surplus proportions were 24% in Hong'an County and 30% in Langzhong and Fushun County, even after the second round reimbursement in 2006, indicative of implementation policies that may discourage making appropriate payments for needed out-of-county care.

We observed two other important reasons for insufficient payments. Some participants reported that they did not know how to obtain reimbursement, and some old people living alone in the countryside did not know how to use the NCMS. The NCMS was started in Fushun in 2005 and in Langzhong and Hong'an in 2006. Changes are said to be forthcoming, and in the near future, patients will not need to pay the full cost in advance, but their copayment [30].

### Policy implication

China is experiencing the largest in-country rural-to-urban migration in history. Data from National Bureau of Statistics of China in 2009 showed that China had 145 million rural-to-urban migrants, and the number has been increasing yearly [31]. Young and healthy people leave their hometown for better job opportunities and higher income in urban and industrial centers, leaving elders and children at home. Migration may contribute to improving household finances, which in turn will improve the ability to join the NCMS. However, the policy is not fair to migrants. Although current NCMS policy requires the enrollment unit to be a household, tending to avoid adverse selection in the near term, continuing barriers to "portability" likely will lead to a decline in enrollment in the longer term as greater numbers continue to leave rural counties across China.

Migrant workers work and live in the city, making it difficult for them to go back to seek medical care, which results in a low utilization of the NCMS. Theoretically, the NCMS should extend its benefits to migrants. In reality, however, the opportunities to sustainably increase the financial protection offered to NCMS enrollees are limited by the financial pressures on local governments [29]. Poor rural counties have very limited resources to support coverage of health care in expensive urban hospitals. This raises a question of whether the cities where migrants move to work should contribute towards insuring against medical costs.

Even though the Urban Employee Basic Medical Insurance in some cities will cover students whose parent(s) legally work in the city, it seldom covers migrant workers themselves [32]. In some cities, such as Guangzhou and Chengdu, local governments are developing projects to recruit rural-to-urban migrant workers into the Urban Employee Basic Medical Insurance. While this approach may hold promise, it may have the unintended consequence of further undermining the financial structure of the NCMS by withdrawing the contributions from economically and healthily better-off migrants from the rural contribution pool. Chengdu, the capital of Sichuan Province, is also piloting an approach that seeks to integrate health insurance for rural and urban residents. This collaborative method, with possible regional applicability, may not be easily generalized in light of the diverse management and benefit plans across the country. In addition, not all provinces have an economically robust 'anchor city' such as Chengdu. Our results underscore the need for further study and careful planning regarding how to best assure adequate health insurance for migrant workers, and ultimately, universal coverage for the entire country. No doubt, this will be a formidable challenge.

### Limitations

We are very much aware that this was a cross-sectional study, and it cannot be used to attribute causal relations. To understand the impact of rural-to-urban migration on household economic status, and on NCMS coverage and utilization, longitudinal research designs will be needed. Other variables, such as type of disease and seriousness of disease, are important factors for household economic status and NCMS utilization. However, we did not have enough information to evaluate their influence, which may result in underestimate mis-estimation of the associations.

## Conclusion

This study featured several noteworthy elements. It showed both a high enrollment rate in the NCMS and a low reimbursement rate for those who received in-patient services, especially among migrants. This low level of support from the NCMS-in practice, failing to increase the financial protection of rural-to-urban migrants-may negatively influence future participation of households with migrants. In some cities, local government has initiated pilot projects integrating health insurance systems integration, but the benefits of this kind of integration will be very limited in a short term due to small scale and the diverse management systems across the country.

In sum, the future of China's dynamic migrant worker economy will depend, in part, on the future of health insurance plans. Portability is one critical feature. Moreover, "health security" likely will be an essential element when fostering worker security and retention. As China continues its evolution toward a more highly trained workforce, one with ever-greater technical sophistication, there will be increasing attention to "human capital", and the adequacy of health insurance will serve as a key foundation stone.

## Competing interests

The authors declare that they have no competing interests.

## Authors' contributions

PQ and YY participated in conduct of the study, data analysis and manuscript drafting. JZ participated in the project design, co-ordination and conduct of the study. XM was the principal investigator of the package 3 and package 4 of the project in China, and participated in the protocol development, project design, conduct of the study, and revision of drafts of the manuscript.

All authors read and approved the final manuscript.

## Appendices

### Appendix A

We exploited a range of 11 variables of household assets (Clock/watch, television, phone/mobile, electric fan, VCD/DVD, sofa, furniture, walls made of grass, clay or adobe, floor made of clay, age of house, and floors of the house), which could be classified into two groups: housing characteristics, and ownership of household durable and semi-durable assets. Most variables were dichotomous having a value of either zero or one. Variables that were not dichotomous such as materials used in housing construction were changed into a dichotomous character, permanent or non-permanent materials of housing construction.

### Appendix B

A common method to extract principal components is to select components where the associated eigenvalue is greater than one. However, it is assumed that the first principal component is a measure of economic status [33]. McKenzie [34] considered the use of additional principal components in characterizing household economic status and concluded that only the first principal component was necessary for measuring wealth. In addition, Filmer and Pritchett pointed out that the factor scores for each variable were difficult to interpret if considering the use of additional components [22]. In previous studies, the first principal component accounted for a range from 12% to 27% of total variation, and in our study, the first principal component accounted for 30.14%. The percentage is not high, which may reflect the complexity of correlations between variables, as each included variable may have its own determinant other than SES [35].

## Pre-publication history

The pre-publication history for this paper can be accessed here:

http://www.biomedcentral.com/1471-2458/11/520/prepub
